# Multidisciplinary Innovation in Neurosurgery and Neuroscience: Advancing Frontiers in Diagnosis, Therapy, and Neurological Rehabilitation

**DOI:** 10.3390/brainsci15090926

**Published:** 2025-08-27

**Authors:** Delia Cannizzaro, Roberto Stefini, Kenan Arnautovic, Franco Servadei

**Affiliations:** 1 Department of Neurosurgery, ASST Ovest Milano Legnano Hospital, Legnano, 20025 Milan, Italy; delia.cannizzaro@gmail.com (D.C.);; 2 Semmes Murphey Clinic and Department of Neurosurgery, University of Tennessee, Memphis, TN 38163, USA; kenanarnaut@yahoo.com; 3 Global Neurosurgery Programme Fondazione IRCCS Istituto Neurologico Carlo Besta, 20133 Milan, Italy

In recent years, neurosurgery and clinical neuroscience have undergone a profound transformation, driven by an increasingly interdisciplinary approach that integrates technological innovation, the refinement of therapeutic protocols, and novel rehabilitative paradigms [1]. These advances are reshaping the management of complex neurological disorders and promoting the development of more effective, less invasive, and highly personalized treatment strategies [2].

This Special Issue of *Brain Sciences* presents nine original contributions that collectively illustrate the breadth and depth of these transformative innovations. The studies featured span topics from minimally invasive spinal surgery and advanced neurosurgical training to intraoperative fluorescence-guided resection, nanomedicine-based drug delivery, and invasive brain–computer interfaces (BCIs), offering a multifaceted yet cohesive vision of the future of neurosurgery.

The adoption of 3D navigation systems is enhancing surgical outcomes by minimizing reliance on traditional instruments, paving the way for more precise and less invasive procedures. In the context of spinal surgery, Bielecki et al. (Contribution 1) demonstrated the clinical utility of the Single-Step Pedicle Screw System (SSPSS) combined with three-dimensional neuronavigation. By eliminating the need for a K-wire and standardizing optimal screw trajectories, the authors achieved a high accuracy rate (95%) in pedicle screw placement and a significant reduction in intraoperative complications. This approach represents a substantial evolution toward safer and more reproducible minimally invasive spinal procedures. In the same way, intraoperative CT-based navigation significantly improves the safety and precision of posterior fixation in congenital craniovertebral junction anomalies, enabling tailored surgical strategies that avoid neurovascular injury and eliminate the need for more invasive hardware, thus representing a meaningful advancement in complex spinal neurosurgery (Contribution 2).

The use of Virtual iMRI in combination with intraoperative imaging represents a promising advance in brain tumor surgery. Grasso et al. (2025) evaluated the elastic image fusion (EIF) method, which combines preoperative MRI with intraoperative CT to better detect residual tumors during glioblastoma surgery. Virtual iMRI showed high sensitivity (100%) but lower specificity (50%), while intraoperative CT had lower sensitivity (56%) but perfect specificity (Contribution 3).

The field of medical education has also embraced immersive technologies. Guerrini et al. (2024) report that the use of augmented reality (AR) and hands-on simulation significantly improves early neurosurgical training. Virtual learning environments not only increase student engagement, they also enhance preoperative technical skills in a controlled, risk-free setting, bridging the gap between theoretical knowledge and clinical competence (Contribution 4).

Fluorescence-guided surgery continues to refine the precision of brain tumor resections. Alomari et al. (2024) investigated the intraoperative use of sodium fluorescein (SF) in vestibular schwannoma surgery, showing that it facilitates extensive tumor resection while minimizing perioperative morbidity. This technique represents a valuable adjunct for maximizing oncological clearance while preserving neurological function (Contribution 5).

New small devices are also being employed for applications in the operating room: non-penetrating titanium clips represent an effective and safe alternative to traditional sutures for dural closure in intradural spinal surgeries, significantly reducing cerebrospinal fluid leak rates and preserving dural integrity, while also minimizing operative time and imaging artifacts (Contribution 6).

In neurovascular surgery, the emergence of new endovascular devices has enabled the treatment of increasingly complex aneurysms. Jee et al. (2024) highlight the clinical efficacy of flow-diverting stents, reporting high aneurysm occlusion rates and a progressive reduction in periprocedural complications. These findings underscore the importance of technical maturation and patient-specific evaluation in the management of cerebrovascular pathologies (Contribution 7).

Robotic-assisted surgery represents another transformative frontier in neurosurgical practice, particularly in the domain of microsurgical precision. Four major robotic platforms—Symani, Da Vinci, ZEUS, and MUSA—widely used in surgical practice were explored across 48 studies involving vascular, lymphatic, and neural anastomoses. While the initial procedural times were longer than those for manual techniques, the analysis demonstrated a clear trend toward improved efficiency as surgical teams progressed along the learning curve. Importantly, the review highlights the potential for robotic systems to enhance neurovascular procedures, particularly in delicate tasks such as microsuturing for bypass surgery [3,4].

A key contribution by Khan et al. (2025) presents a scoping review on invasive BCIs aimed at restoring communication in patients with severe motor deficits, such as those resulting from ALS, brainstem stroke, or high cervical spinal cord injury. Recent advances in intracortical neural decoding—translating brain signals into text or synthesized speech—offer a therapeutic frontier with the potential to restore communicative autonomy in otherwise locked-in patients. These innovations represent a foundational milestone in neurotechnological rehabilitation (Contribution 8).

Nanotechnology is also opening up new therapeutic pathways in neuro-oncology. Khilar et al. (2025) reviewed strategies employing engineered nanoparticles for the targeted delivery of chemotherapeutic, immunotherapeutic, and radiotherapeutic agents. By enhancing blood–brain barrier permeability and enabling combined treatment modalities (e.g., radio-immunotherapy), these nanocarriers hold great promise for overcoming existing pharmacologic limitations. However, the transition to clinical application necessitates further investigations into long-term safety and nanotoxicology (Contribution 9).

Collectively, these studies demonstrate that neurosurgery is no longer an isolated specialty but rather a convergence point for biomedical engineering, computational neuroscience, advanced simulation-based education, and translational oncology. Emerging technologies—from neuronavigation and intraoperative fluorescence to BCIs and nanomedicine—are not only improving clinical outcomes but fundamentally redefining the paradigms of diagnosis, intervention, and rehabilitation.

To consolidate these promising advances and ensure their widespread adoption, future efforts must prioritize multicenter prospective trials, regulatory validation, and economic sustainability. Nevertheless, the momentum in this field is undeniable.

Neurosurgery and neuroscience are entering a phase of rapid transformation. The integration of advanced technologies—from implantable devices and AR simulators to precision nanotherapeutics—is enhancing not only therapeutic interventions but also medical training and patients‘ quality of life. Innovations such as flow diversion and fluorescein-guided surgery place surgical precision and safety at the heart of clinical progress, while immersive simulation is revolutionizing professional training. In parallel, invasive BCIs offer a tangible hope of restoring essential functions such as communication in patients otherwise rendered voiceless. Nanomedicine, meanwhile, appears poised to overcome longstanding pharmacological barriers in the treatment of brain tumors, although robust clinical validation remains essential. These findings underscore the value of robotic technologies in extending the boundaries of technical feasibility and precision in cranial neurosurgery and merit further clinical validation and the integration of robotics into advanced neurovascular interventions.

Artificial intelligence is rapidly emerging as a pivotal tool in neurosurgery, enhancing diagnostic accuracy, surgical planning, and intraoperative decision-making through advanced data analysis and machine learning algorithms. Predictive models developed using AI can stratify patient risk, anticipate surgical outcomes, and personalize treatment strategies, promoting evidence-based precision neurosurgery [5,6,7].

Ultimately, the synergy between technology, surgery, and neuroscience holds the potential to transform complex neurological disease management, from optimizing minimally invasive procedures to revolutionizing specialist training and pioneering new oncologic and rehabilitative strategies. This multidisciplinary convergence, anchored in both technological innovation and clinical insights, promises a future in which neurosciences will not only advance therapies but fundamentally reshape our understanding and treatment of neurological disease.

In reflecting on this progress, it is worth recalling the metaphor coined by Bernard of Chartres in the 12th century, often invoked in medicine, to acknowledge our intellectual debt to the giants on whose shoulders we stand: our earlier inspirations. While that legacy remains foundational, it is increasingly complemented by the transformative role of emerging technologies, which continue to empower us to elevate the standard of care for our patients.
